# From Sea Sponge to Clinical Trials: Starting the Journey of the Novel Compound PM742

**DOI:** 10.3390/md22080339

**Published:** 2024-07-26

**Authors:** Patricia G. Cruz, Rogelio Fernández, Raquel Rodríguez-Acebes, Marta Martínez-Díez, Gema Santamaría-Núñez, Marta Pérez, Carmen Cuevas

**Affiliations:** Research and Development, PharmaMar S.A., Pol. Ind. La Mina Norte, Avda. de los Reyes 1, 28770 Colmenar Viejo, Spain; rfernandez@pharmamar.com (R.F.); rrodriguez@pharmamar.com (R.R.-A.); mmdiez@pharmamar.com (M.M.-D.); gsnunez@pharmamar.com (G.S.-N.); mperez@pharmamar.com (M.P.); ccuevas@pharmamar.com (C.C.)

**Keywords:** sponge, *Discodermia du Bocage*, cytotoxicity, isolation, structural elucidation, tubulin interaction

## Abstract

PM742 (**1**), a new chemical entity, has been isolated from the sponge *Discodermia du Bocage* collected in the Pacific Ocean. This compound showed strong in vitro cytotoxicity against several human tumor cell lines as well as a tubulin depolymerization mechanism of action, which led us to conduct an extensive Structure-Activity-Relationship study through the synthesis of different analogs. As a result, a derivatively named PM534 (**2**) is currently in its first human Phase I clinical trial. Herein, we present a comprehensive review of the isolation, structural elucidation, and antitumor activities of the parent compound PM742.

## 1. Introduction

**Figure 1:**
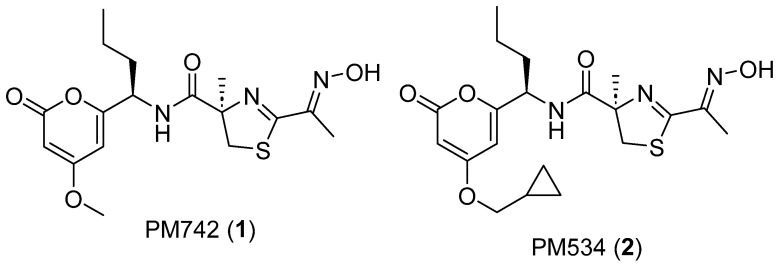
Chemical structures of PM742 (**1**) and PM534 (**2**).

Marine sponges of the genus *Discodermia* have proven to be an enormously rich source of secondary metabolites with interesting biological activities. Among the most significant compounds, discodermolide revealed immunosuppressive and cancer killing properties [1]. Additionally, discodermins [2] possess antimicrobial and anticancer activities, while calyculins [3] have proven to be strong serine/threonine protein phosphatase inhibitors. In our continuing efforts to isolate anticancer compounds from marine sources, we have investigated the chemical composition of a *Discodermia du Bocage* specimen collected off the coast of Halmahera, the largest island in the Maluku Islands (Indonesia). The chemical chromatographic profile of this sample revealed the presence of major aurantoside-type structures such as aurantoside E. However, a different peak present in the sample caught our attention, leading us to fractionate this sample. As a result of this study, we discovered the new compound PM742 (**1**) (Figure 1).

The planar structure of **1** was elucidated by a combination of spectrometric and spectroscopic methods including High Resolution Mass Spectrometry (HRMS) and extensive ^1^H, ^13^C, and 2D Nuclear Magnetic Resonance (NMR). The absolute configuration of the stereogenic centers was determined by Marfey´s analysis [4]. In particular, we used the synthetic methods of chemical degradation and derivatization, along with the chemical synthesis of different amino acid enantiomers and comparison with those present in the natural molecule. PM742 is a new nonribosomal peptide–polyketide metabolite [5,6] containing a unique trisubstituted dihydrothiazole moiety and a 4-methoxy α-pyrone group. 

The biological activity of **1** against a panel of eight different human cancer cell lines was also evaluated. Strong in vitro cytotoxicity was found for this compound. Furthermore, preliminary in vitro studies suggested that PM742 is a tubulin depolymerization agent. These features prompted us to initiate a drug development program, confirming its structure by total synthesis, which will be described in a separate publication. Moreover, another synthetic analog of this compound, PM534 (**2**) (Figure 1), provided a novel microtubule destabilizing agent, with an optimized interaction for the colchicine site and a novel mechanism of action, with a high tubulin-binding affinity [7]. Accordingly, Phase I clinical trials have been initiated with compound **2** for cancer treatment.

## 2. Results and Discussions

### 2.1. Bioactivity-Guided Fractionation of the Organic Extract of the Sponge and Isolation of **1**

After extracting the chopped sponge with a mixture of 1:1 CH_2_Cl_2_/CH_3_OH, the crude organic extract was initially subjected to chromatographic separation using vacuum liquid chromatography (VLC). Subsequently, the produced VLC fractions were tested for their cytotoxic potential against four human cancer cell lines: A-549 (lung carcinoma); HT-29 (colorectal carcinoma); MDA-MB-231 (breast adenocarcinoma); and PSN-1 (pancreatic adenocarcinoma). The cytotoxic activity was observed for the fraction eluted with H_2_O/CH_3_OH 1:3, which had a reddish color due to the presence of aurantosides. This fraction was subjected to semipreparative HPLC to afford a purer fraction H5, but still with an orangish color. Thus, H5 was further subjected to semipreparative chromatographic separation to isolate 14.1 mg of a white powder corresponding to compound **1**.

### 2.2. Structure Elucidation of **1**

The molecular formula of **1** was established using (+)HRESTOFMS as C_17_H_24_N_3_O_5_S based on the molecular ion parent at *m*/*z* 382.1429 [M+H]^+^ (calcd. for C_17_H_24_N_3_O_5_S 382.1431). The IR spectrum of **1** showed absorption bands at 3277, 1718, 1686, and 1647 cm^−1^, indicating the presence of hydroxy, ester, oxime, and amide groups. The analysis of ^1^H and ^13^C NMR data in combination with HSQC obtained in CD_3_CN (Table 1) revealed the presence of four methyl groups (one of them methoxy), three methylenes (sp^3^), three methines (one sp^3^ and two sp^2^), seven quaternary carbons (one sp^3^ and six sp^2^), and two interchangeable protons at 7.07 and 9.73 ppm. The puzzle of all these signals was solved through COSY and HMBC correlations observed in the corresponding experiments. 

**Figure 2:**
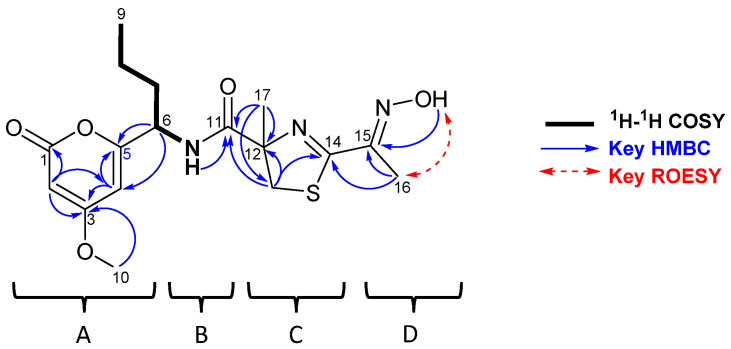
**COSY** (bold), HMBC (blue), and ROESY (red) correlations for PM742.

Analysis of 2D experiments of **1**, including COSY and HMBC, allowed us to detect the presence of three substructures **A**–**D**. (Figure 2, Figures S3 and S5).

Long-range correlations observed in HMBC spectrum from H-2 (δ_H_ 5.41) to carbons C-1, C-3, and C-4 (δ_C_ 164.5, 172.1, and 99.7), from H-4 (δ_H_ 5.89) to carbons C-2, C-3, C-5, and C-6 (δ_C_ 88.7, 172.1, 165.1, and 51.6) and from H_3_-10 (3.78) to C-3 (δ_C_ 172.1) allowed us to establish the presence of a methoxy α-pyrone polyketide residue as fragment A. The methine at C-6 (δ_H_ 4.66; δ_C_ 51.6) showed proton and carbon shifts characteristic of an α-amino group, and COSY correlations allowed us to identify the spin system from N-H (δ_H_ 7.07) to H_3_-9 (δ_H_ 0.94), characteristic of norvaline amino acid residue, as fragment B. The third fragment was proposed starting from the isolated spin system corresponding to the methylene at position C-13 (δ_H_ 3.54, 3.17; δ_C_ 40.7), its HMBC correlations to the quaternary carbons C-11, C-12, and C-14 (δ_C_ 174.8, 85.3, and 168.6) and from H_3_-17 (δ_H_ 1.48) to C-11, C-12, and C-13 (δ_C_ 174.8, 85.3, and 40.7), which indicated the presence of a 4-methyl-2-thiazoline ring as fragment C. Protons from the remaining methyl singlet CH_3_-16 (δ_H/C_ 2.17/11.3) showed a HMBC correlation with the quaternary carbon C-15 (δ_C_ 153.7). But the HMBC spectrum acquired in CD_3_OH showed two clear cross-peaks between C-15 (δ_C_ 152.8) and H_3_-16 (δ_H_ 2.18), as well as the interchangeable proton O-H (δ_H_ 11.82), suggesting the presence of a ketoxime as fragment D.

Finally, the four identified fragments A, B, C, and D were joined using key HMBC correlations from H-6 (δ_H_ 4.66) to C-4 and C-5 (δ_C_ 99.7 and 165.1), from N-H (δ_H_ 7.07) and H_3_-17 (δ_H_ 1.48) to C-11 (δ_C_ 174.8), and from H_3_-16 (δ_H_ 2.17) to C-14 and C-15 (δ_C_ 168.6 and 153.7), allowing us to propose the planar structure of **1**, as shown in Figure 2. The geometry of the oxime was determined as *E* based on ROESY spectrum acquired in CD_3_OH showing a clear cross-peak between H_3_-16 (δ_H_ 2.18) and O-H (δ_H_ 11.82). Although there are some examples of marine-derived molecules incorporating 4,6-disubstituted-4-methoxy-α-pyrone moiety [8,9], methylated thiazoline [10,11], norvaline residue [12,13], or oxime functional group [14,15], compound **1** is the first example of an α-pyrone polyketide fused to a peptide fragment containing an oxime residue. The antibiotic althiomycins [16] of microbial origin similarly contain an aldoxime group on a 4-thiazolicarboxamide moiety, but still differ greatly from **1**.

**Figure 3:**
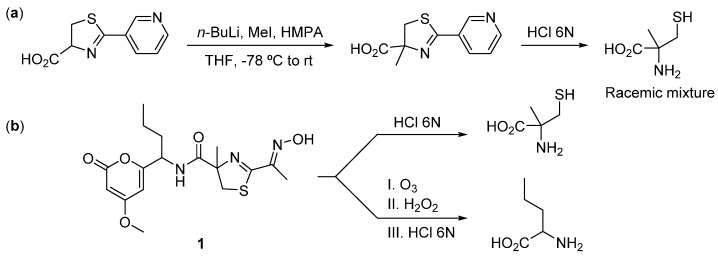
(**a**) Preparation of 2-methylcysteine. (**b**) Hydrolysis and ozonolysis of **1**.

The absolute configuration of the stereogenic centers was determined by Marfey’s analysis. Compound **1** was hydrolyzed in acidic media and then derivatized with *L*-FDAA. The norvaline residue could not be obtained by simple hydrolysis, and the compound was therefore first subjected to oxidative ozonolysis before subsequent hydrolysis. Since only 2-methyl-*L*-cysteine was commercially available, it was necessary to prepare the corresponding enantiomer to compare the relative chromatographic retention times of both with the amino acid present in the natural molecule [17] (Figure 3). Comparison of the natural sample with derivatized amino acids of known stereochemistry allowed for the unambiguous assignment of the stereogenic centers. These studies confirmed the presence of 2-methyl-*L*-cysteine and *D*-norvaline in **1** (Figures S14 and S15).

### 2.3. Cytotoxicity and Antiproliferative mechanism

#### 2.3.1. Cytotoxicity Assays

The in vitro cytotoxic activity of the new compound (Table 2) was tested against eight human tumor cell lines, breast (MDA-MB-231), colon (HT-29 and LoVo), lung (A-549 and NCI-H460), ovary (A-2780 and IGROV-1), and pancreas (PSN-1) following a published procedure [18]. Colchicine was used as the positive control. Compound **1** exhibited strong activity with GI_50_ values in the submicromolar range.

#### 2.3.2. Mechanism of Action

**Figure 4:**
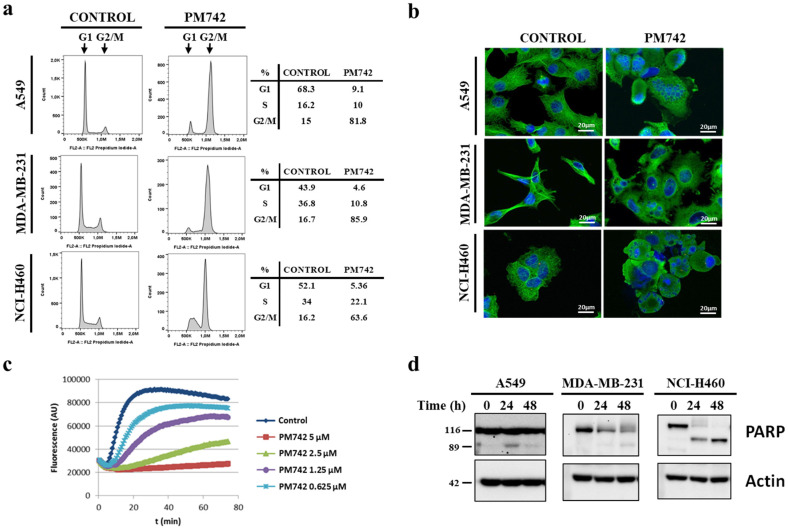
PM742 arrests cells in the G2/M phase and induces apoptosis by inhibiting tubulin polymerization in human tumor cells. (**a**) A-549, MDA-MB-231, and NCI-H460 cells were treated with 100 nM PM742 for 24 h, fixed, permeabilized, and chromatin stained with PI. DNA content was analyzed using flow cytometry. (**b**) A-549, MDA-MB-231, and NCI-H460 cells were treated with 100 nM PM742 for 24 h and then washed, fixed, and stained with an anti-α-tubulin primary antibody and an Alexafluor 488 secondary antibody to visualize the tubulin cytoskeleton. Nuclei were counterstained with Hoechst 33342. Pictures were taken with a fluorescence microscope. (**c**) Purified porcine brain tubulin was incubated at 37 °C in the presence or absence of the indicated concentrations of PM742 for 80 min. Aliquots of the reaction mix were taken at 1 min intervals to follow the dynamics of polymerization. (**d**) A-549, MDA-MB-231, and NCI-H460 cells were treated with 100 nM PM742 for 24 or 48 h, and lysates were obtained and analyzed using Western blot with the appropriated primary and secondary antibodies. Parp cleavage by caspases is widely accepted as indicative of apoptosis.

To unveil the antiproliferative mechanism of **1**, we initially assessed its impact on the cell cycle of tumor cells (A-549, MDA-MB-231, and NCI-H460) using flow cytometry with propidium iodide (PI) DNA staining. Following a 24 h exposure to 100 nM PM742, a significant accumulation of cells in G2/M phase was observed (Figure 4a). Furthermore, we evaluated the tubulin cytoskeleton using immunofluorescence microscopy. A-549, MDA-MB-231, and NCI-H460 cancer cells lines were treated with **1** for 24 h, followed by staining of microtubules using a primary anti-α-tubulin and secondary AlexaFluor 488 antibodies. In control cells, the microtubule network exhibited a well-organized structure. However, treatment with 100 nM PM742 resulted in a noticeable cytoskeleton disorganization, likely attributed to tubulin polymerization inhibition (Figure 4b). Consequently, these results indicated that PM742-mediated inhibition of microtubule dynamics leads to in cell cycle arrest at the G2/M transition phase. The accumulation of cells in the mitotic phase results from the inability to form the mitotic spindle, essential for the separation of the sister chromatids into the two daughter cells, a classical phenotypic effect of tubulin depolymerization agents. Therefore, we investigated whether **1** could inhibit tubulin polymerization in vitro with a fluorescence assay with pig brain tubulin as substrate. As shown in Figure 4c, **1** at the concentration of 1.25 µM inhibited nearly 50% of tubulin polymerization after 40 min, with the complete blockage observed at 5 µM. Furthermore, since mitotic arrest triggers cell death, we examined whether **1** induces apoptosis. To this end, we checked PARP processing using Western blot, a canonical marker of apoptosis, using a PARP-specific antibody directed against the C-terminal region, which recognizes both the complete protein and the fragment generated by caspase-mediated proteolytic cleavage during apoptosis. Treatment with **1** led to a decrease in the complete PARP protein (116 kDa) and an increase in the cleaved protein in all the cell lines analyzed, although with varying kinetics (Figure 4d). Clear PARP cleavage was observed in NCI-H460 and A-549 cells at 24 h, with a more potent effect in the former, while 48 h of treatment was required to observe PARP fragmentation in MDA-MB-231, indicating that the antiproliferative effect of **1** on these cells is due to the induction of apoptosis. Overall, these results suggest that the antiproliferative action of **1** arises from G2/M phase arrest, mitotic cell accumulation, and subsequent apoptosis induction. Furthermore, PM742’s antimitogenic and apoptotic effects are attributable to its interaction with microtubules.

## 3. Materials and Methods

### 3.1. General

^1^H and ^13^C NMR were recorded on a Varian Unity 500 spectrometers at 500 MHz and 125 MHz, respectively. Chemical Shifts (δ) are reported in parts per millions (ppm) referenced to CD_3_OD at δ_H_ 3.30 ppm for ^1^H and δ_C_ 49.0 ppm for ^13^C and to CD_3_CN at δ_H_ 1.93 ppm for ^1^H and δ_C_ 1.3 ppm for ^13^C. Coupling constants are reported in Hertz (Hz), with the following abbreviations used: s = singlet, d = doublet, t = triplet, q = quartet, and m = multiplet. Optical rotations were determined using a Jasco P-1020 polarimeter with a sodium lamp and are reported as follows: [α]^25^_D_ (*c* g/100 mL, solvent). High Resolution Mass Spectroscopy (HRMS) was performed using an Agilent 6230 TOF LC/MS system (spectrometer employing 0.04% of formic acid in H_2_O and CH_3_CN as ionic mobile phase). The (+)ESIMS were recorded using an Agilent 1100 Series LC/MSD spectrometer. IR spectra were obtained with a Perkin-Elmer Spectrum 100 FT-IR spectrometer with ATR sampling. UV spectra were performed using a Cary 60 2.0 UV−VIS spectrometer (*c* mg/mL, solvent).

### 3.2. Animal Material

A sponge of the order Lithistida, family Theonellidae, genus *Discodermia du Bocage*, 1869 was collected by hand using a rebreather diving system in Halmahera, Indonesia (2° 16.307′ N/127° 44.466′ E), at depths ranging between 6 and 73 m. The animal material was identified by Dr. María Jesús Uriz (Centre for Advanced Studies of Blanes). A sample of the specimen was deposited at the Centre for Advanced Studies of Blanes in Girona, Spain, with the reference code HALM-706.

### 3.3. Extraction and Isolation of **1**

The frozen specimen (104 g) was diced and extracted at room temperature under magnetic stirring, firstly with a mixture of 1:1 CH_2_Cl_2_/CH_3_OH (3 × 500 mL) and later with H_2_O (1 × 300 mL). Organic and aqueous extracts were evaporated to provide dry residues of 5.3 g and 213 mg, respectively. The organic extract was subjected to step gradient VLC on Lichroprep RP-18 from H_2_O to CH_3_OH and subsequently from CH_3_OH to CH_2_Cl_2_. The fraction eluted with H_2_O/CH_3_OH 1:3 (80.4 mg) was subjected to semipreparative reversed phase HPLC (XBridge C18, 5 μm, 10 × 150 mm, isocratic H_2_O + 0.04% TFA/CH_3_CN + 0.04% TFA (65:35) for 3 min, gradient from 35 to 60% CH_3_CN + 0.04% TFA in 19 min and from 60 to 100% CH_3_CN + 0.04% TFA in 3 min, UV detection, flow 3 mL/min). Fraction H5, with a retention time of 14.2 min, was subsequently purified using semi-preparative reversed phase HPLC (SymmetryPrep C18, 7 μm, 7.8 × 150 mm, isocratic H_2_O + 0.04% TFA/CH_3_CN + 0.04% TFA (75:25) for 3 min, gradient from 25 to 80% CH_3_CN + 0.04% TFA in 18 min, UV detection, flow 2.8 mL/min) to afford **1** (14.1 mg, retention time 11.3 min).

Compound **1**: White powder. (+)ESIMS: *m*/*z* 382.3 [M + H]^+^, 404.1 [M + Na]^+^, 785.2 [2M + Na]^+^, 157.2 [C_6_H_9_N_2_OS]^+^, 181.2 [C_10_H_13_O_3_]^+^; (+)HRESTOFMS: *m*/*z* 382.1429 [M+H]^+^ (calcd for C_17_H_24_N_3_O_5_S 382.1431, Δ = 0.52 ppm); ^1^H (500 MHz) and ^13^C NMR (125 MHz) see Table 1. [α]^25^ _D_ = +89.8 (0.5018 g/100 mL, CHCl_3_). UV (CH_3_OH) λ_max_ (log ε) = 228 nm (4.20). FTIR spectra (cm^−1^): 3277, 2960, 1718, 1686, 1647, 1566, 1509, 1452, 1409, 1245, 1024, 907, 819.

### 3.4. Synthesis of 2-methylcysteine 

Since *L*-methylcysteine is the only commercially available stereoisomer of this amino acid, we had to prepare the racemic mixture to obtain an adequate standard to later compare with the natural one. We followed the process described by Singh et al. [17]. *n*-Butyllithium (0.41 mL, 1.6 M in hexane, 0.65 mmol) was added dropwise to a stirred solution of 2-pyrid-3-yl-4,5-dihydro-1,3-thiazole-4-carboxylic acid (55 mg, 0.26 mmol) in anhydrous THF (5 mL) at −78 °C under N_2_ atmosphere. The red mixture obtained was stirred at that temperature for 1.5 h. A solution of iodomethane (97 μL, 1.56 mmol) and hexamethylphosphoramide (0.28 mL, 1.56 mmol) in dry THF (1 mL) was added, and the color of the mixture changed to orange. After an additional 1.5 h at −78 °C and 1.5 h at room temperature, the reaction was quenched at 0 °C with an aqueous saturated solution of NH_4_Cl. The organic phase was decanted, and the aqueous layer was extracted thoroughly with EtOAc. The combined organic solutions were dried over anhydrous Na_2_SO_4_, filtered, and evaporated to dryness. Purification of the crude residue using semipreparative HPLC (Pursuit XRs C18, 5 μm, 10 × 150 mm, eluting with MeOH/H_2_O, gradient from 40% to 64% MeOH in 12 min) afforded 1.7 mg of the expected racemic methylated ester. Acid hydrolysis with 1 mL of 6N HCl in a sealed vial at 110 °C for 16 h yielded the deprotected amino acid ready for Marfey’s derivatization. 

### 3.5. Absolute Configuration of the Amino Acid Residues in **1**

In total, 0.3 mg of **1** were dissolved in 0.5 mL of 6N HCl in a sealed vial and heated at 110 °C for 16 h. The solvent was evaporated under a N_2_ stream, the residue was dissolved in 50 μL of H_2_O, and 0.5 mg of fluorodinitrophenyl-5-*L*-alaninamide (*L*-FDAA, Marfey’s reagent) in 100 μL of acetone and 40 μL of 1N aqueous NaHCO_3_ were added. The resulting mixture was heated at 40 °C for 1 h and, after cooling to room temperature, neutralized with 100 μL of 2N HCl. Finally, the mixture was diluted with 800 μL of H_2_O and filtrated (45 μm filter) prior to HPLC-MS analysis. 

Synthetic racemic mixture of *DL*-2-methyl-cysteine and commercial *L*-2-methyl-cysteine were derivatized in the same manner as the hydrolyzed compound **1**. Retention times of the derivative amino acid standards, were determined using reversed phase HPLC-MS: Symmetry C18, 5 mm, 4.6 × 150 mm, gradient H_2_O + 0.04% TFA/CH_3_CN + 0.04% TFA from 20% to 50% CH_3_CN + 0.04% TFA in 30 min, UV (215 and 350 nm) and (+)ESIMS detection, flow 0.8 mL/min. Comparison of these retention times (*D* and *L*-MeCys derivatized, t_R_: 5.66 min, and 6.30 min, respectively) with the natural amino acid residue (t_R_: 6.39 min) unambiguously confirmed the presence of 2-methyl-*L*-cysteine in compound **1**.

The norvaline residue could not be obtained by simple hydrolysis of **1**, and therefore the compound was first subjected to an oxidative ozonolysis step. For this, a stream of ozone in O_2_ was bubbled through a cooled solution of **1** (0.3 mg) in CH_2_Cl_2_ (3 mL) at −78 °C for 5 min. The solvent was evaporated under a N_2_ stream, and the residue was dissolved in 2 mL of hydrogen peroxide (35%): formic acid (1:9) at 0 °C and kept for 2 h. Then, the solvent was removed under a stream of N_2_; the resultant residue was hydrolyzed in acidic conditions and immediately subjected to Marfey’s derivatization as described above. Finally, it was analyzed using HPLC-MS using the same conditions described previously for the non-oxidized sample. Comparison of the retention times of authentic standards of *L*- and *D*-norvaline derivatized (t_R_: 25.33 and 21.21 min, respectively) with the amino acid residue of the natural sample (t_R_: 25.33 min) confirmed the presence of *D*-norvaline in compound **1**.

### 3.6. Biological Activity

#### 3.6.1. In Vitro Assays

The aim of this assay is to evaluate the in vitro cytostatic (ability to delay or arrest tumor cell growth) or cytotoxic (ability to kill tumor cells) activity of the samples being tested. A colorimetric assay, using sulforhodamine B (SRB) reaction has been adapted to provide a quantitative measurement of cell growth and viability [19,20]. This form of assay employs 96-well cell culture microplates. Cell lines derived from different types of human cancer used in this study were obtained from the American Type Culture Collection (ATCC): A-549 (ATCC CCL-185), lung carcinoma; HT-29 (ATCC HTB-38) and LoVo (ATCC CCL-229) colorectal carcinoma; MDA-MB-231 (ATCC HTB-26), breast adenocarcinoma; and PSN-1 (ATCC CRL-3211), pancreatic adenocarcinoma [21]. Ovarian adenocarcinoma A-2780 and IGROV-1 cell lines were obtained from the National Cancer Institute and kindly gifted by Dr. D’Incalci (Mario Negri Institute, Italy), respectively. Cell lines were maintained in Dulbecco’s Modified Eagle Medium (DMEM) (for A-549, HT-29, MDA-MB-231, NCI-H460, and LoVo) or RPMI (for PSN-1, A-2780, and IGROV-1), supplemented with 10% Fetal Bovine Serum (FBS), 2 mM L-glutamine, 100 U/mL penicillin, and 100 U/mL streptomycin, at 37 °C, 5% CO2, and 98% humidity. For the experiments, cells were harvested from subconfluent cultures using trypsinization and resuspended in fresh medium before counting and plating.

Cells were seeded in 96-well microtiter plates, at 5 × 10^3^ cells per well in aliquots of 150 μL and allowed to attach to the plate surface for 18 h (overnight) in drug-free medium. After that, one control (untreated) plate of each cell line was fixed (as described below) and used for time zero reference value. Culture plates were then treated with test compounds (50 μL aliquots of 4× concentrated compound stock solutions made in complete culture medium) using ten serial dilutions (concentrations ranging from 1 to 0.000262 μg/mL) and triplicate cultures (final concentration of DMSO being 1%). After 72 h treatment, the antitumor effect was measured by using the SRB methodology, as follows: Briefly, cells were washed twice with PBS, fixed for 15 min in 1% glutaraldehyde solution at room temperature, rinsed twice in PBS, and stained in 0.4% SRB solution for 30 min at room temperature. Cells were then rinsed several times with 1% acetic acid solution and air-dried at room temperature. SRB was then extracted in 10 mM trizma base solution, and the absorbance was measured in an automated spectrophotometric plate reader at 490 nm. Results are expressed as GI_50_, the concentration that causes 50% inhibition in cell growth after correction for cell count at the start of the experiment, by applying the NCI algorithm [21]. Doxorubicin, colchicine, and DMSO (solvent) were used as the positive and negative controls in this assay. Prism 3.03 from GraphPad was used for the statistical analysis of the cell growth inhibition results. Using the mean ± SD of triplicates, a dose–response curve was automatically generated using nonlinear regression analysis to a four-parameter logistic curve. Three reference parameters were calculated (NCI algorithm) by automatic interpolation: GI_50_ = compound concentration that produces 50% cell growth inhibition, as compared to control cultures; TGI = total cell growth inhibition (cytostatic effect), as compared to control cultures; and LC_50_ = compound concentration that produces 50% net cell killing (cytotoxic effect).

#### 3.6.2. Inhibition of Tubulin Polymerization In Vitro

Tubulin polymerization assays were performed using a fluorescence-based Tubulin Polymerization kit (Cytoskeleton Inc, Denver, CO, USA) following the manufacturer’s instructions. Briefly, purified porcine brain tubulin was incubated with the tubulin reaction mix in the presence or absence of **1** in 96-well plates. Then, tubulin polymerization was initiated by transferring the plate to a 37 °C chamber of a plate reader. Polymerization is followed by fluorescence enhancement due to the incorporation of a fluorescent reporter into microtubules as polymerization occurs. The polymerization dynamics of tubulin were monitored for 80 min at 37 °C by measuring the change in fluorescence every 1 min using a Perkin Elmer Victor3 fluorimeter, at excitation of 355 nm, and emission of 460 nm. Fluorescence is directly related to the amount of tubulin polymer present. Typical curves of polymerization include the three phases of microtubule formation: a nucleation phase, a growing phase, and the equilibrium phase.

#### 3.6.3. Immunofluorescence Assay

Cells were treated with the appropriate concentration of **1** for 24 h and then fixed (4% paraformaldehyde), permeabilized (0.5% Triton X-100), and incubated with a blocking solution (5% bovine serum albumin in PBS) for 30 min. Cells were then incubated with primary mouse anti-human α-tubulin antibody for 1 h at room temperature. After three washes with a PBS/BSA 1% solution, cells were incubated with Alexafluor 488-conjugated goat anti-mouse IgG secondary antibody at room temperature for 1 h. After washing, preparations were counterstained with Hoechst 33342, labeling DNA, and mounted with Mowiol mounting medium. Pictures were taken with a Zeiss Axiovert 200M microscope equipped with a 63× oil immersion objective an Axiocam HRm digital camera and an Apotome-1 Structured Illumination System (Zeiss, Jena, Germany).

#### 3.6.4. Cell Cycle Analysis Using Flow Cytometry

For the cell cycle experiments, cells were incubated with 100 nM PM742 or the drug vehicle (DMSO) for 24 h, and then washed with PBS and fixed with 70% cold ethanol. Cells were then treated with RNAse I and stained with 50 µg/mL propidium iodide. Samples were analyzed with a BD Accuri-6 flow cytometer (Becton and Dickinson, Franklin Lakes, NJ, USA) and the FlowJo v10.1 cytometry analysis software (Tree Star, Ashland, OR, USA).

#### 3.6.5. Western Blotting

For immunoblotting, cell protein extracts were prepared following standard procedures in a buffer composed of 20 mM Tris-HCl (pH 7.5), 150 mM, NaCl, 1% (*v*/*v*) Nonidet P-40, and 2 mM EDTA in the presence of protease and phosphatase inhibitors. After quantification with the Micro-BCA Protein Assay Kit (Thermo Scientific), proteins were separated using SDS-PAGE and transferred to PVDF membranes (Immobilon-P, Millipore). Membranes were sequentially probed with primary and appropriate secondary (horseradish-peroxidase-conjugated) antibodies following the manufacturer’s instructions. Antibody–antigen complexes were detected using the ECL system (GE Healthcare, Marlborough, MA, USA) and the ChemiDoc imager (Bio-Rad, Hercules, CA, USA).

## 4. Conclusions

In this work, we present the novel anti-tubulin agent PM742 (**1**), a small molecule that showed efficient antitumoral properties in vitro, and that was isolated from a *Discodermia* sponge from Halmahera, Indonesia. Its structure was determined using extensive 1D and 2D NMR and HRMS. Marfey´s method was applied to fully confirm the absolute configuration of the stereogenic centers present in compound PM742. Compound **1** is the first example of an α-pyrone polyketide fused to a nonribosomal peptide origin fragment containing an oxime residue. 

Our search underscores the importance of perseverance in scientific exploration and highlights the innate instinct of the PharmaMar chemists, since despite encountering initially discouraging results, we were still determined to study this specimen more deeply. PM742 arrests cells in G2/M phase and induces apoptosis by inhibiting tubulin polymerization in human tumor cells. Consequently, this work has resulted in a preclinical program of in vivo experiments. Furthermore, PM534, a synthetic analog of this compound, has initiated Phase I clinical trials. Our findings serve as a testament to the rewards that can be reaped through unwavering determination and a commitment to pushing the frontiers of knowledge.

## 5. Patents

This work has given rise to a new patent application, as follows: Martín, M.J.; Rodríguez-Acebes, R.; Cruz, P.G.; Francesch, A.M.; Cuevas, C. Anticancer Compounds. WO 2020127194, 26 June 2020.

## Figures and Tables

**Table 1:** ^1^H and ^13^C NMR data of **1** in CD_3_CN and CD_3_OD.

NO	CD_3_CN		CD_3_OD	
	δ_C_, Type	δ_H_ (*J* in Hz)	δ_C_, Type	δ_H_ (*J* in Hz)
1	164.5, C	-	166.7, C	-
2	88.7, CH	5.41, d (2.3)	88.9, CH	5.54, d (2.2)
3	172.1, C	-	173.4, C	-
4	99.7, CH	5.89, dd (2.3, 0.5)	100.8, CH	6.04, d (2.1)
5	165.1, C	-	165.2, C	-
6	51.6, CH	4.66, m	52.2, CH	4.75, m
7	35.0, CH_2_	1.78, m	35.3, CH_2_	1.85, m
8	19.8, CH_2_	1.42, m	20.2, CH_2_	1.47, m
		1.34, m		1.39, m
9	13.8, CH_3_	0.94, t (7.4)	13.8, CH_3_	0.99, t (7.4)
10	57.1, CH_3_	3.78, s	57.0, CH_3_	3.84, s
11	174.8, C	-	176.6, C	-
12	85.3, C	-	85.6, C	-
13	40.7, CH_2_	3.54, d (11.6)	40.6, CH_2_	3.54, d (11.5)
		3.17, d (11.6)		3.18, d (11.5)
14	168.6, C	-	170.3, C	-
15	153.7, C	-	152.8, C	-
16	11.3, CH_3_	2.17, s	11.0, CH_3_	2.18, s
17	25.1, CH_3_	1.48, s	25.0, CH_3_	1.53, s
NH	-	7.07, d (8.45)	-	7.84, d (8.7)
OH	-	9.73, s	-	11.82, s *

* Assigned from spectrum acquired in CD_3_OH.

**Table 2:** Cytotoxic activity data (M) of compound **1**.

GI_50_ (M)	Breast	Colon		Lung		Ovary		Pancreas
	MDA-MB-231	HT-29	LoVo	A-549	NCI-H460	A-2780	IGROV-1	PSN-1
**PM742 (1)**	3.97 × 10^−8^	3.03 × 10^−8^	1.60 × 10^−8^	3.03 × 10^−8^	3.82 × 10^−8^	2.50 × 10^−8^	2.80 × 10^−8^	4.42 × 10^−8^
**Colchicine**	1.70 × 10^−8^	1.83 × 10^−8^	4.81 × 10^−8^	6.77 × 10^−8^	3.23 × 10^−8^	1.55 × 10^−8^	3.25 × 10^−8^	3.37 × 10^−8^

## Data Availability

The data are contained within the article or Supplementary Materials.

## Supplementary Materials

The following supporting information can be downloaded at: https://www.mdpi.com/article/10.3390/md22080339/s1, Figures S1–S10: NMR spectra of **1**; Figure S11: (+)HRESITOFMS spectrum of **1**; Figures S12–S15; Marfey´s analysis.
