# Supplementary material for: From Sea Sponge to Clinical Trials: Starting the Journey of the Novel Compound PM742
Source: Mar Drugs. 2024 Jul 26;22(8):339. doi: 10.3390/md22080339 (PMC11355788; doi:10.3390/md22080339)

Supplementary Material

From Sea Sponge to Clinical Trials I: Revealing the Journey of the Novel Compound PM742

Patricia G. Cruz,* Rogelio Fernández, Raquel Rodríguez-Acebes, Marta Martínez-Díez, Gema Santamaría-Núñez, Marta Pérez and Carmen Cuevas

Research and Development, PharmaMar S.A., Avda. de los Reyes 1, Pol. In. La Mina, 28770 - Colmenar Viejo, Madrid, Spain.

*Corresponding autor: *pcruz@pharmamar.com*

Table of contents

1. NMR Spectra of PM742 (1)

Figure S1. ^{1}H NMR spectrum of **PM742**, (500 MHz,CD_3CN)..………...………………….……...**3**
Figure S2. ^{13}C NMR spectrum of **PM742**, (125 MHz, CD_3CN)…...………………………….…...**4**
Figure S3. *g*-COSY spectrum of **PM742**, (500 MHz, CD_3CN)………...…………………….……**5**
Figure S4. *g*-HSQC spectrum of **PM742**, (500 MHz, CD_3CN)……………...…….……………**6**
Figure S5. *g*-HMBC spectrum of **PM742**, (500 MHz, CD_3CN)…………………….………..**7**
Figure S6. ROESY spectrum of **PM742**, (500 MHz, CD_3CN)………………………………......**8**
Figure S7. ^{1}H NMR spectrum of **PM742**, (500 MHz, CD_3OH)….……...………...…….……...**9**
Figure S8. ^{13}C NMR spectrum of **PM742**, (125 MHz, CD_3OH)…....……………..…………...**10**
Figure S9. *g*-HMBC spectrum of **PM742**, (500 MHz, CD_3OH)……………..………...………**11**
Figure S10. ROESY spectrum of **PM742**, (500 MHz, CD_3OH)……………………...…...…...**12**

2. MS spectrum of 1

Figure S11. (+)HRESITOFMS spectrum of **1**…………………………………………………...**13**

3. Marfey´s analysis

Figure S12. (+)ESIMS of hydrolysis of **1**…………………………………………………… **14**
Figura S13. (+)ESIMS of hydrolysis of **1** after ozonolysis……………………………………**15**
Figure S14. Extracted ion chromatogram of L-FDAA derivatives for NorVal………………..…**16**
Figure S15. Extracted ion chromatogram of L-FDAA derivatives for MeCys……….………..…**17**

Figure S1. ^{1}H NMR spectrum of **PM742**, (500 MHz, CD_3CN)

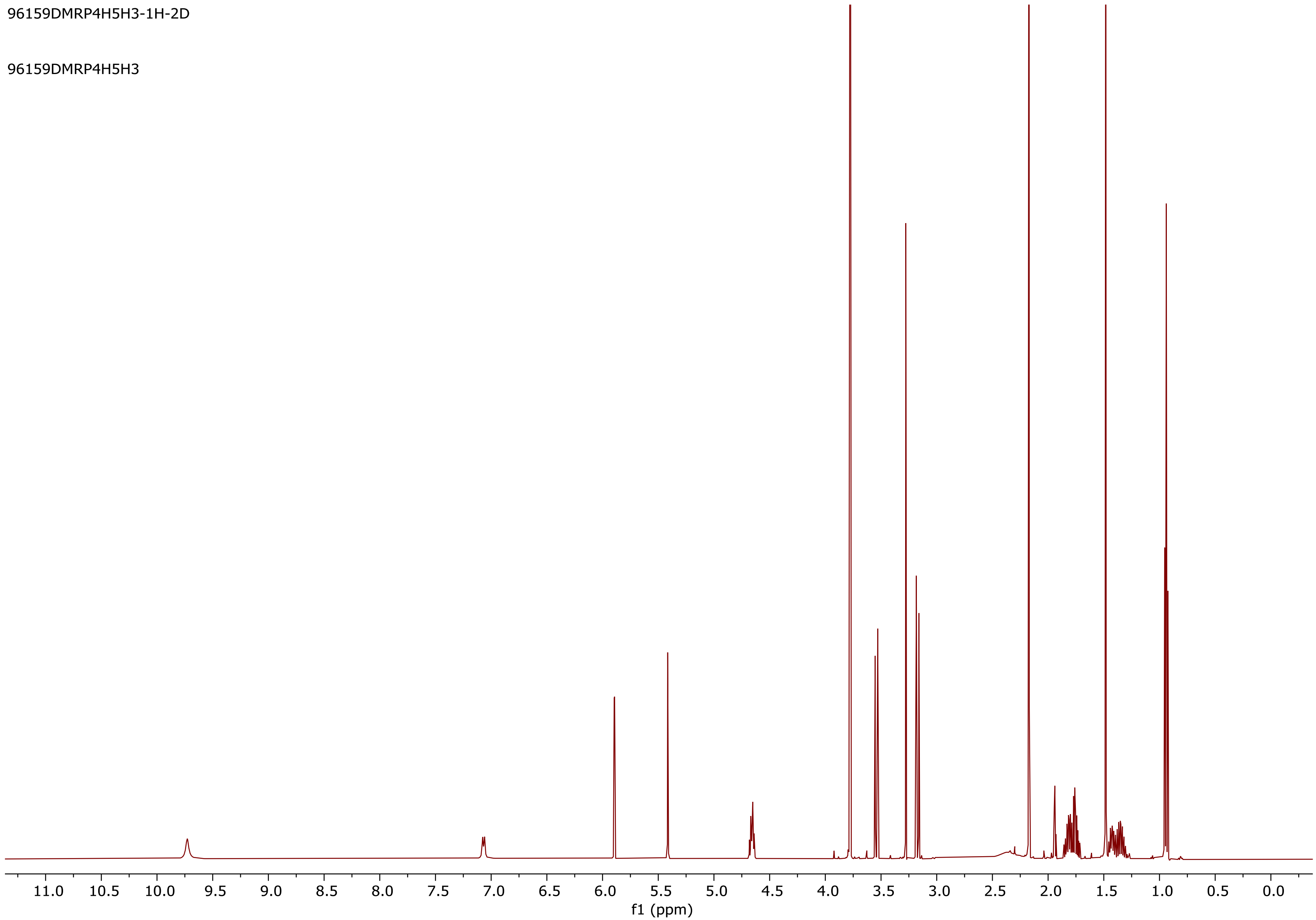

Figure S2. ^{13}C NMR spectrum **PM742**, (125 MHz, CD_3CN)

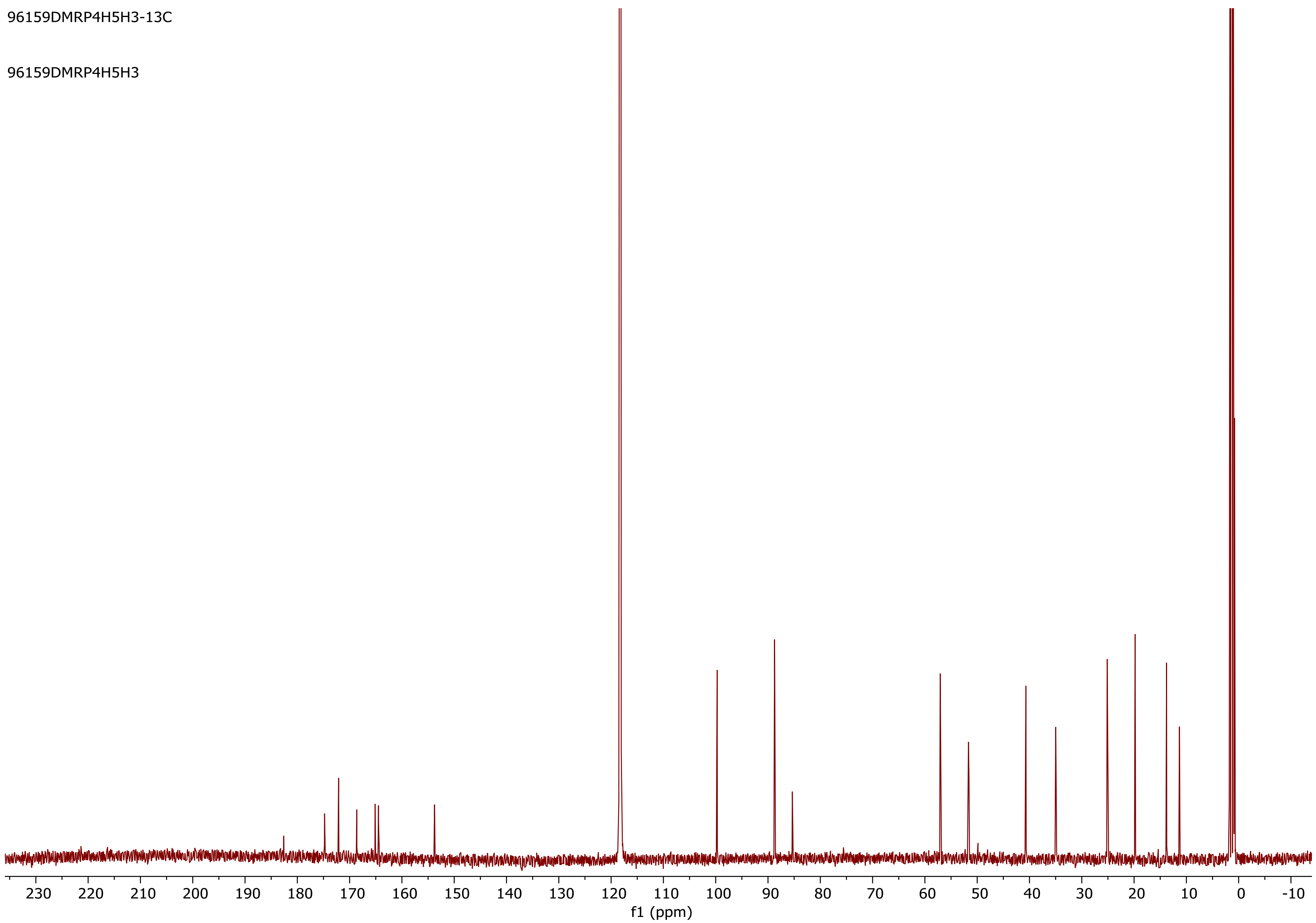

Figure S3. gCOSY spectrum **PM742**, (500 MHz, CD_3CN)

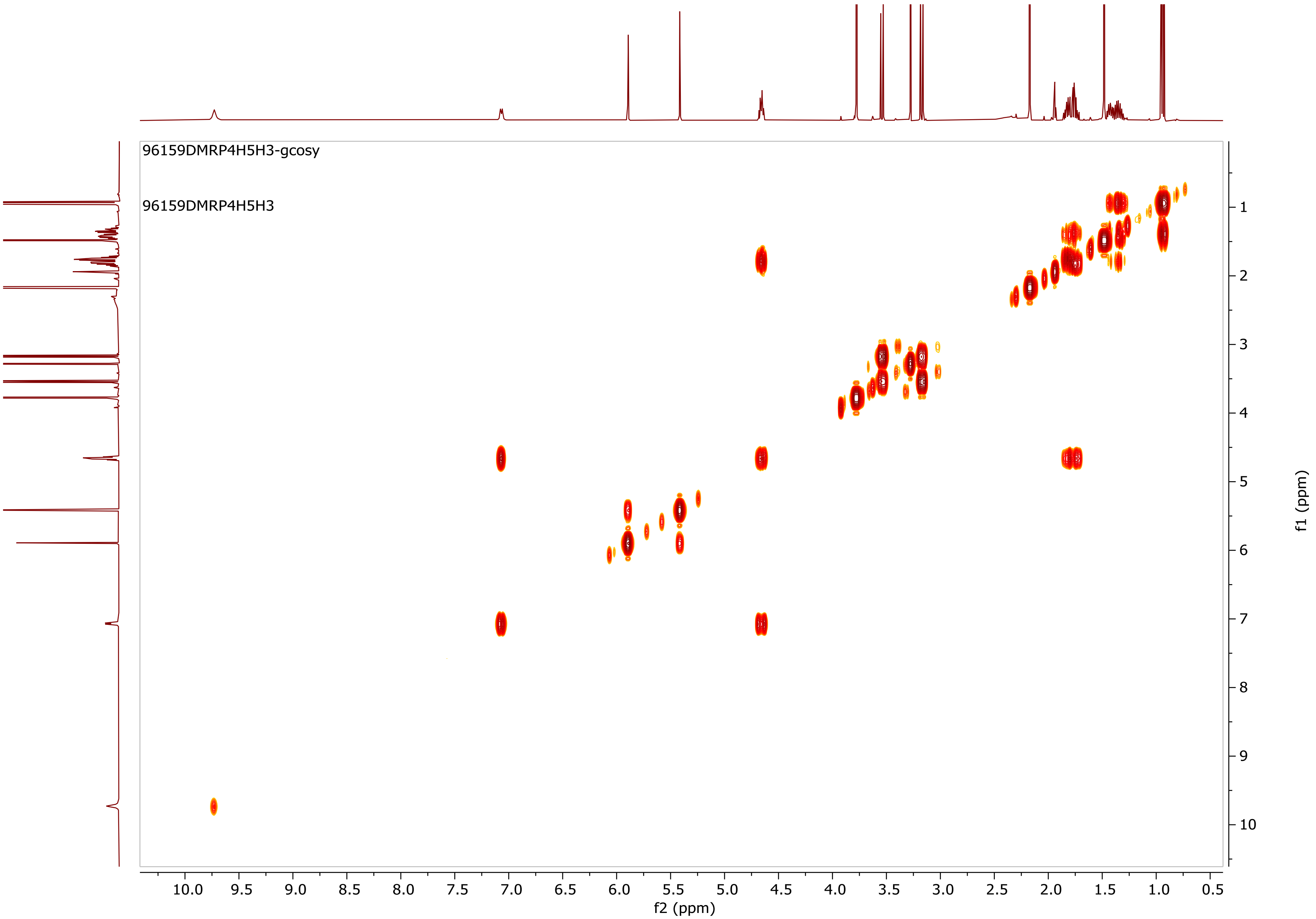

Figure S4. gHSQC spectrum **PM742**, (500 MHz, CD_3CN)

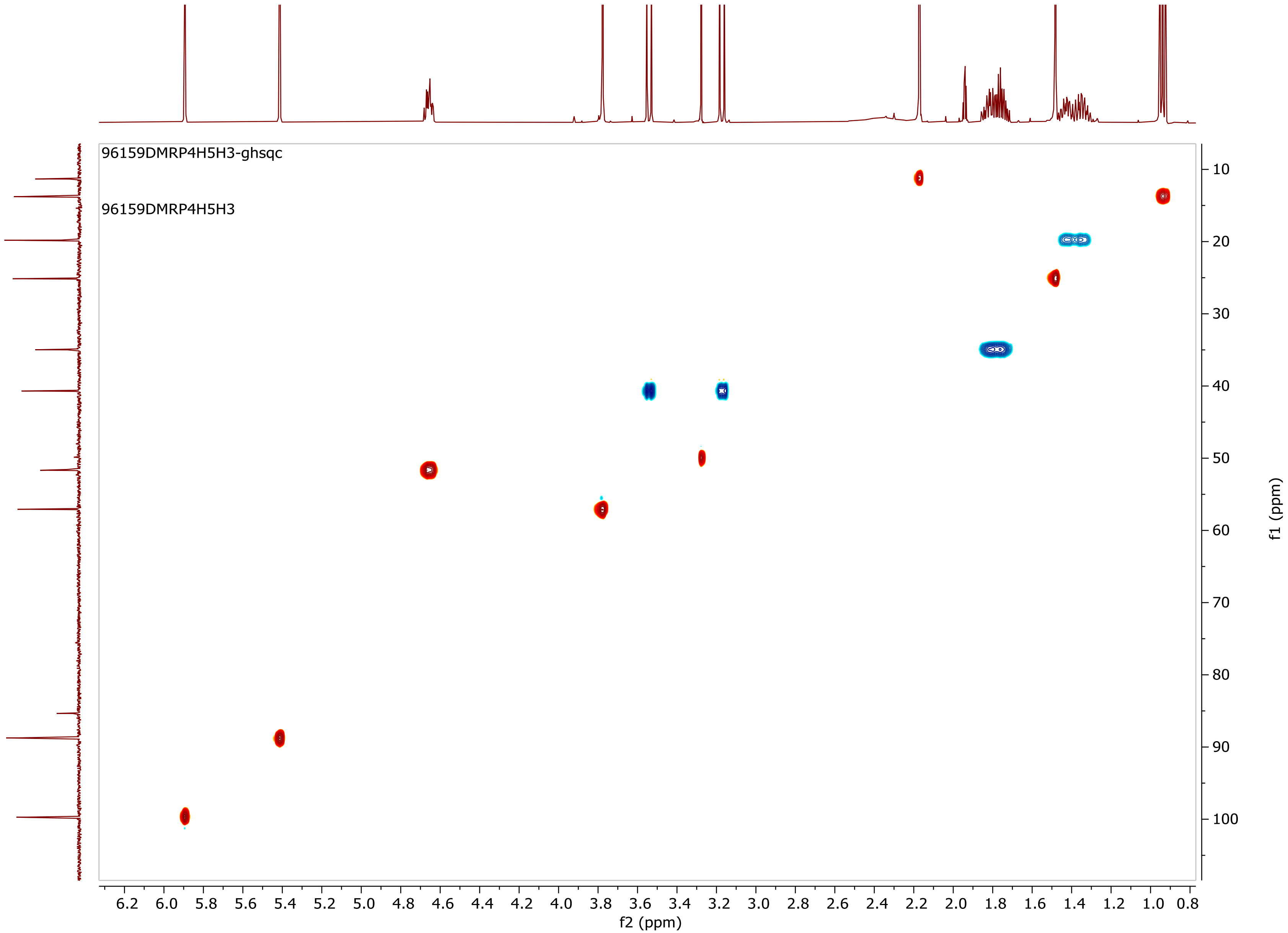

Figure S5. gHMBC spectrum **PM742**, (500 MHz, CD_3CN)

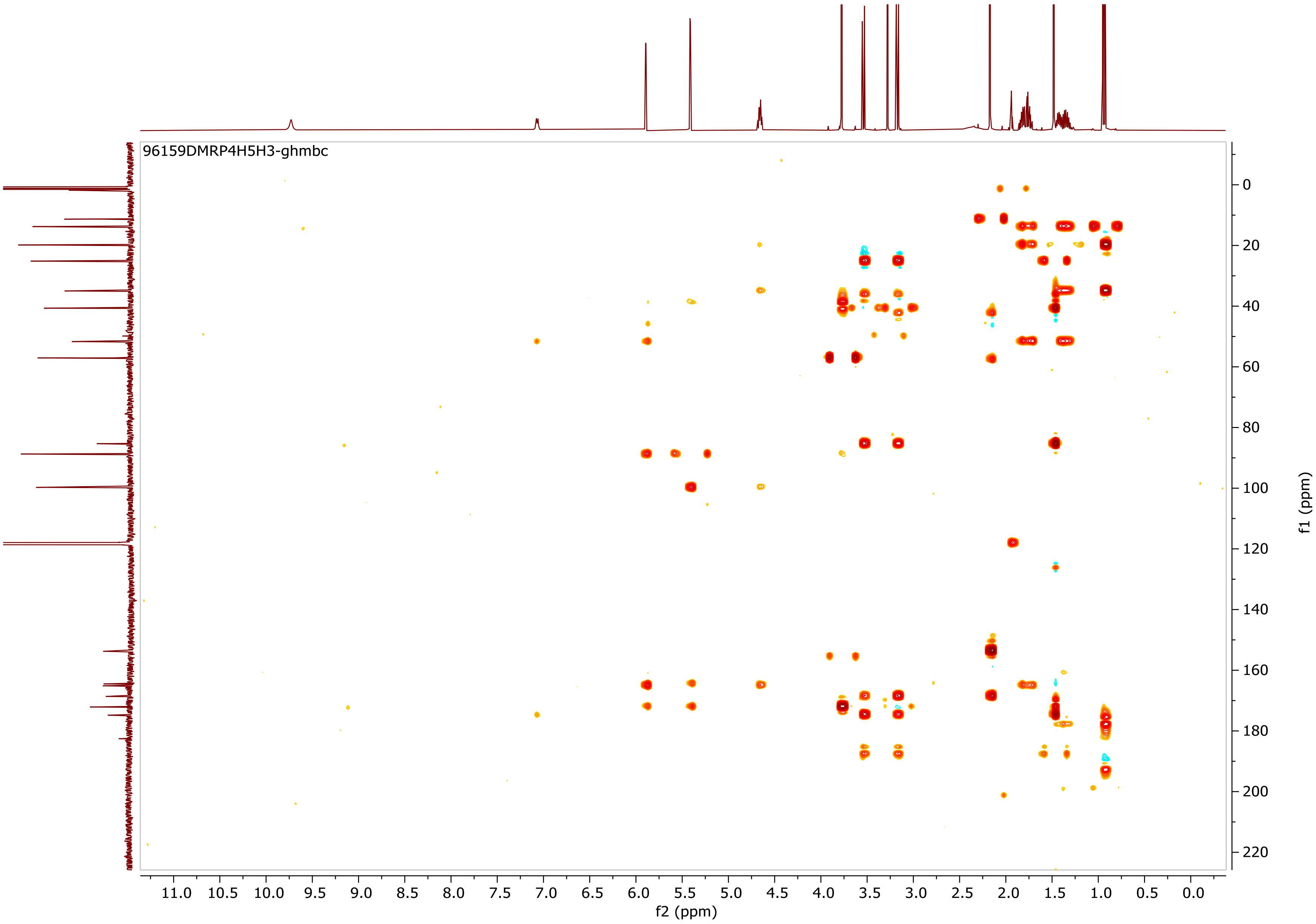

Figure S6. ROESY spectrum **PM742**, (500 MHz, CD_3CN)

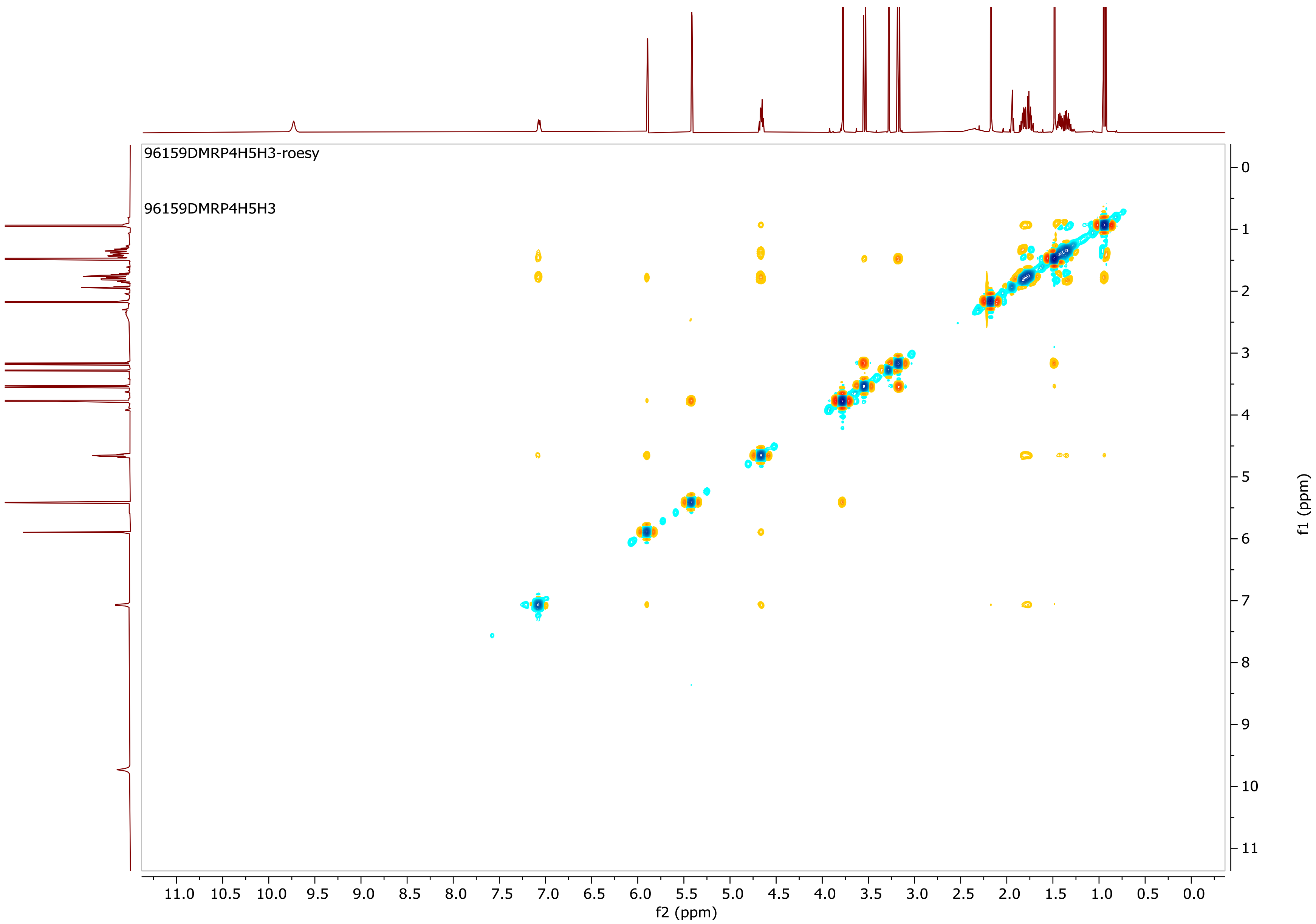

Figure S7. ^{1}H NMR spectrum of **PM742**, (500 MHz, CD_3OH)

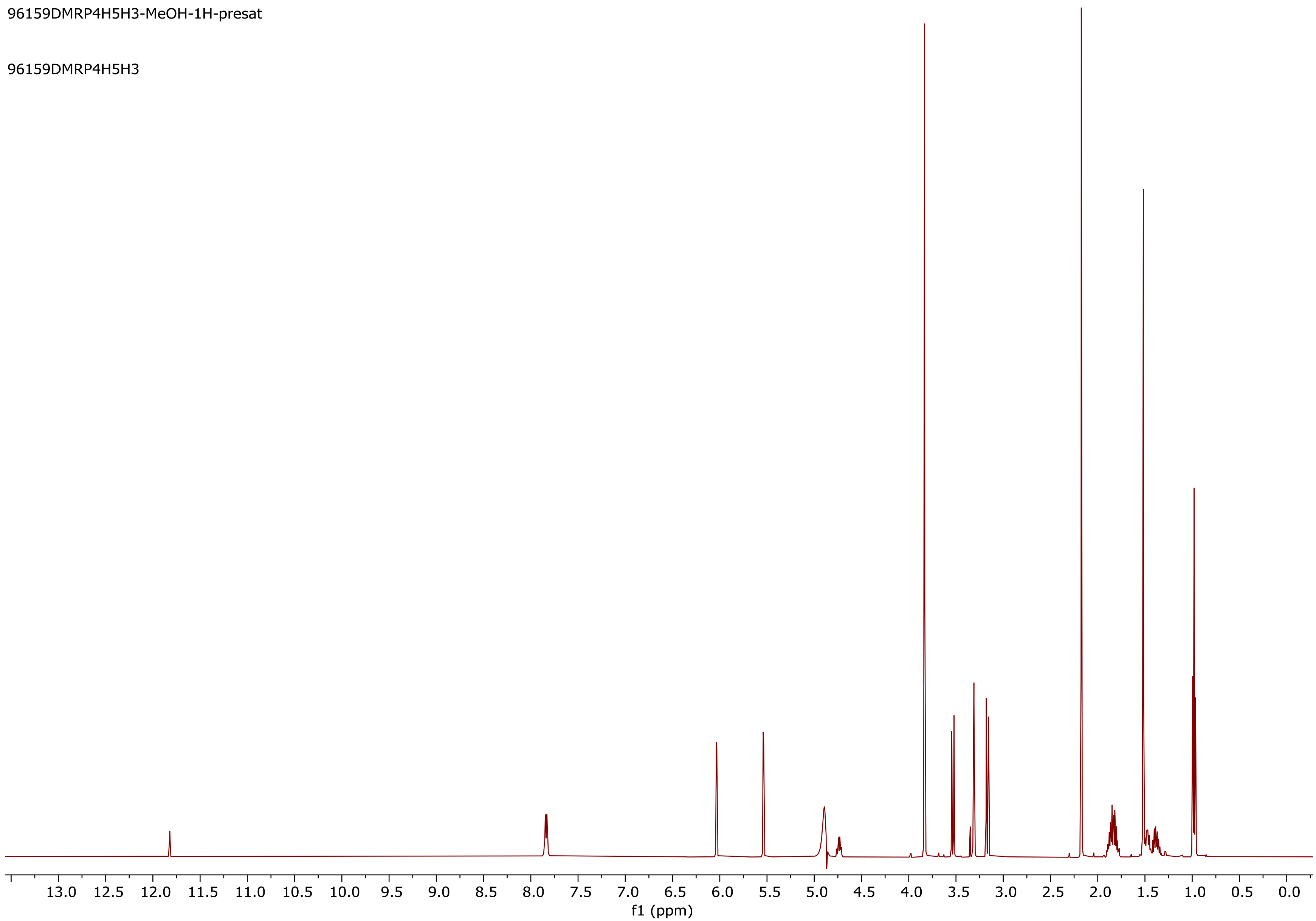

Figure S8. ^{13}C NMR spectrum **PM742**, (125 MHz, CD_3OH)

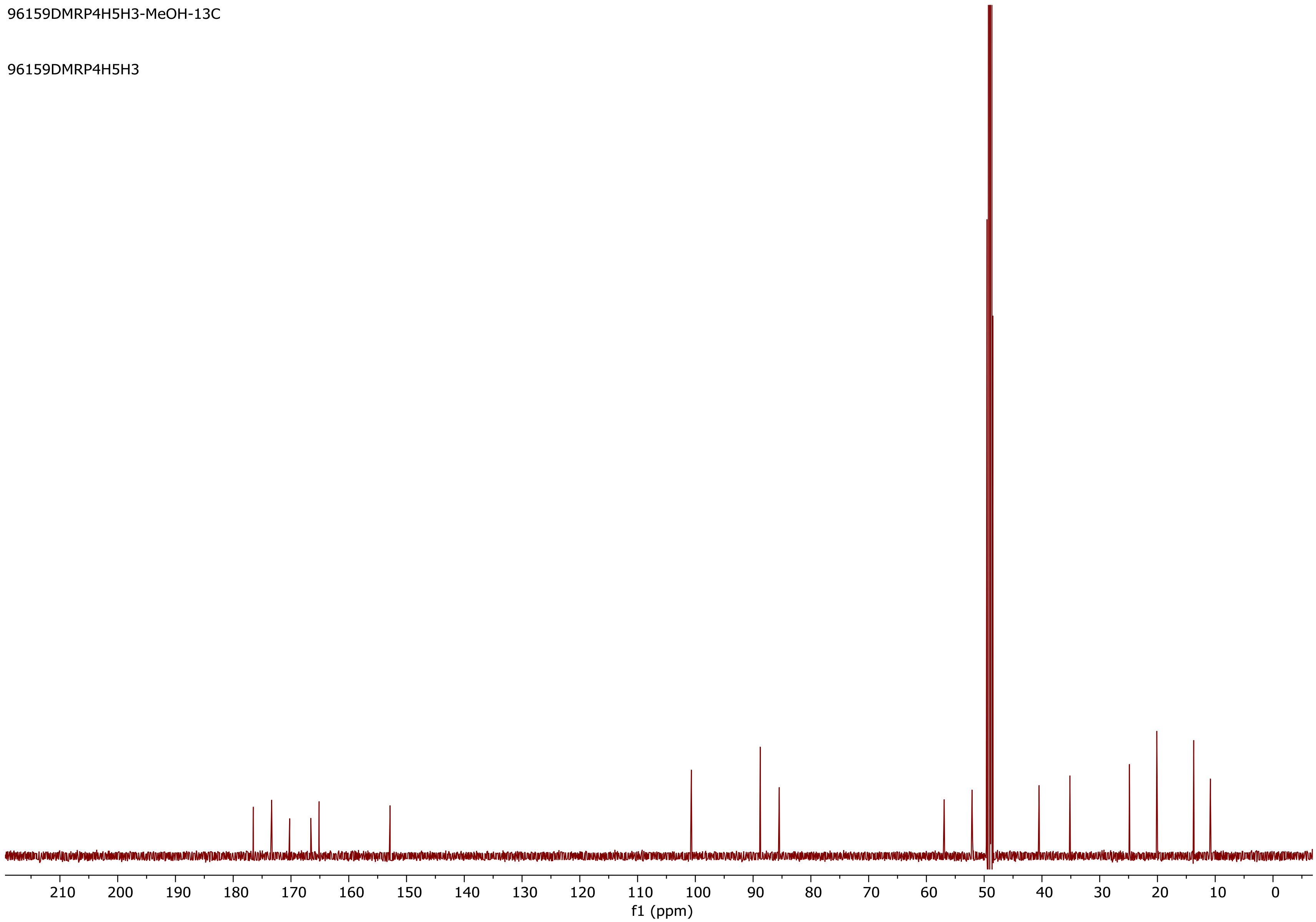

Figure S9. gHMBC spectrum **PM742**, (500 MHz, CD_3OH)

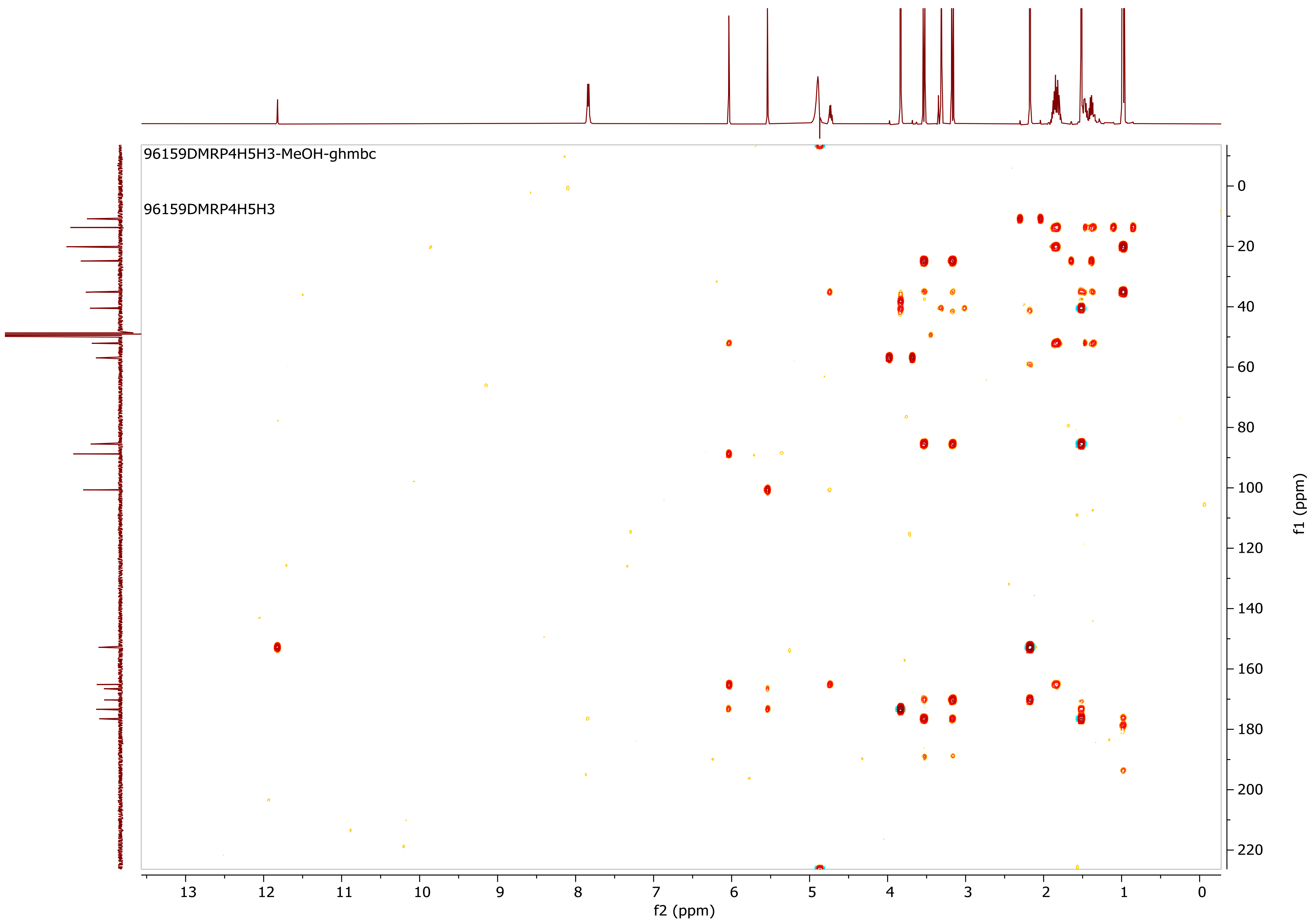

Figure S10. ROESY spectrum **PM742**, (500 MHz, CD_3OH)

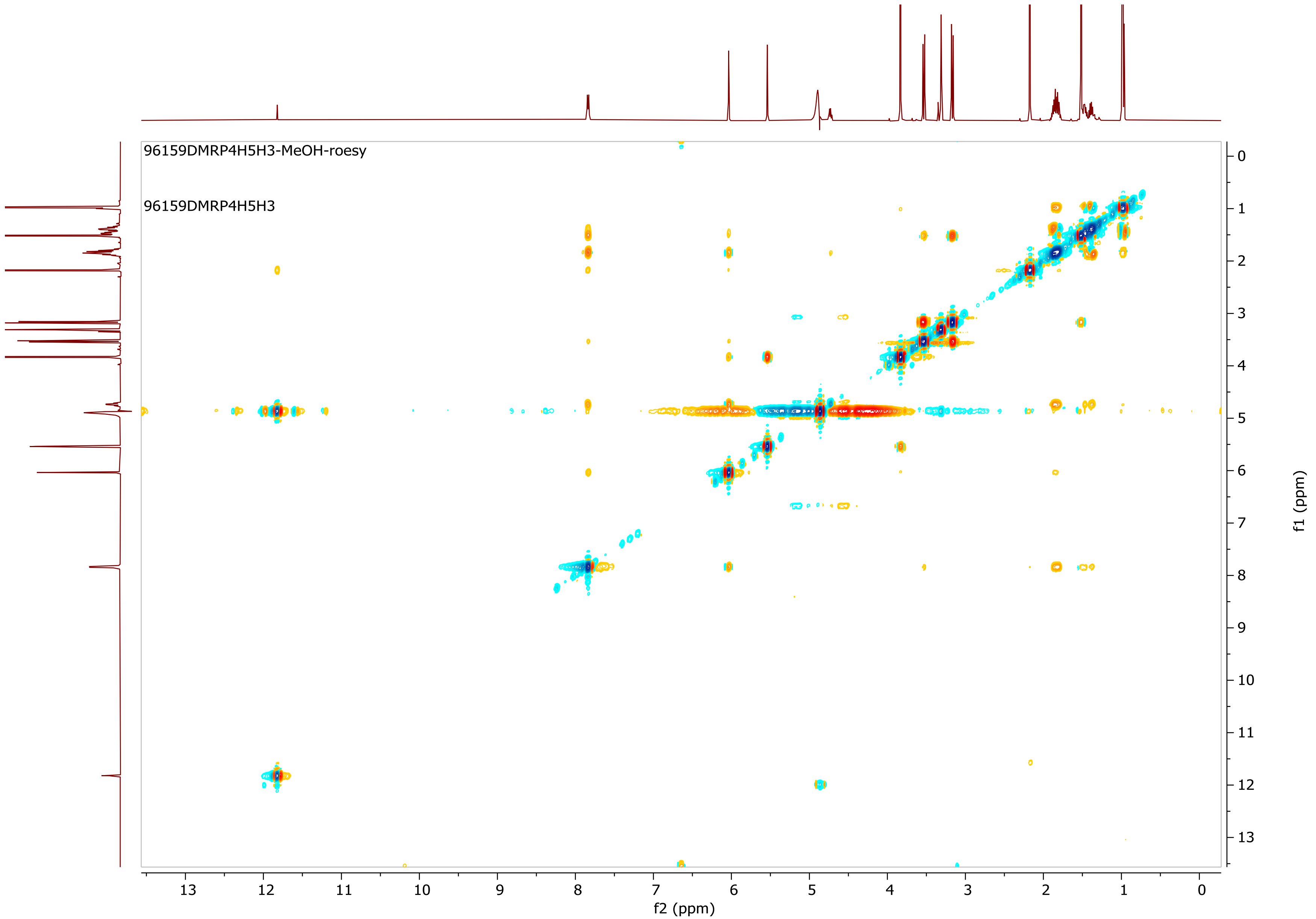

Figure S11. (+)-HRESITOFMS spectrum of **1**

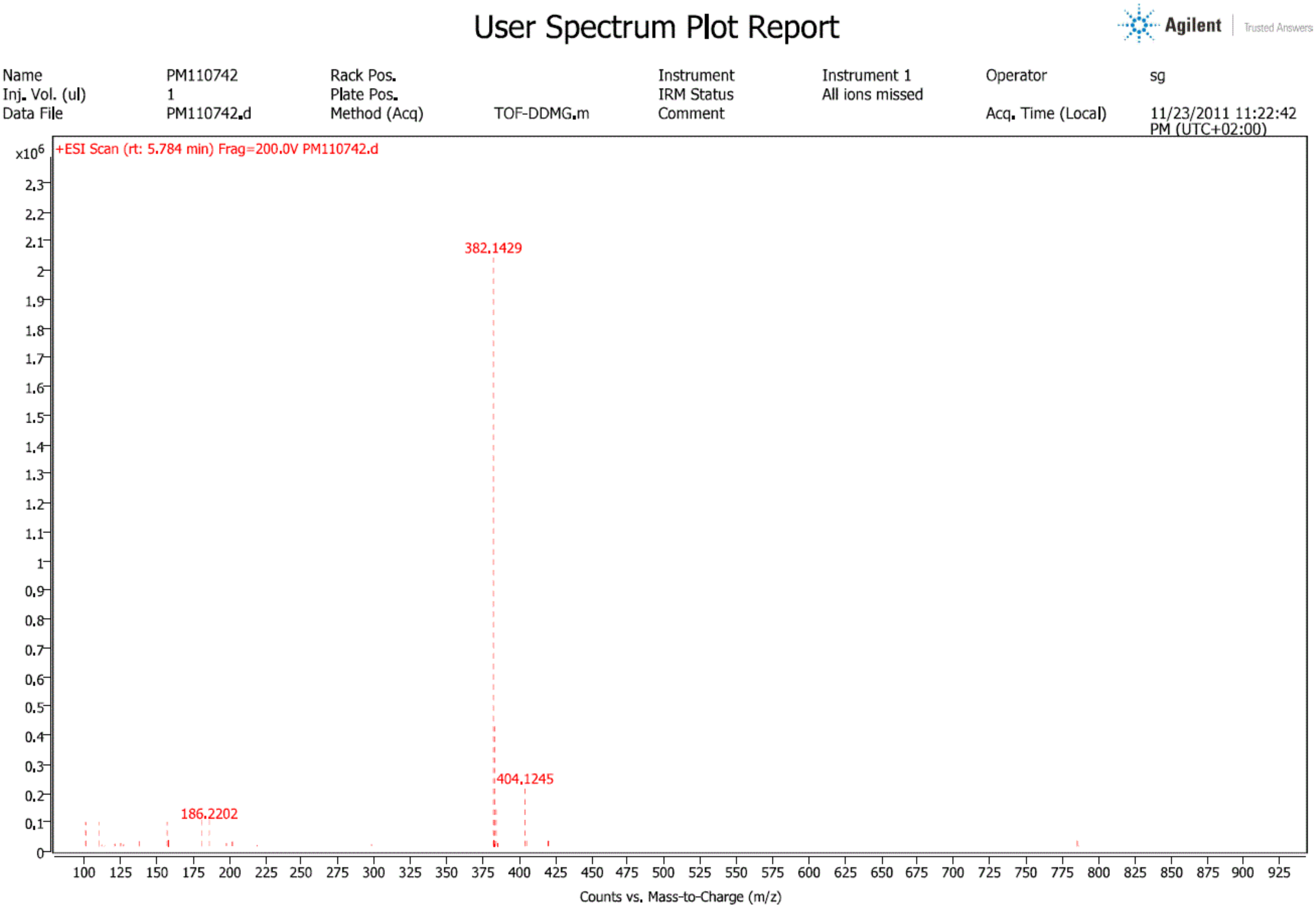

Figure S12. (+)ESIMS of hydrolysis of **1**

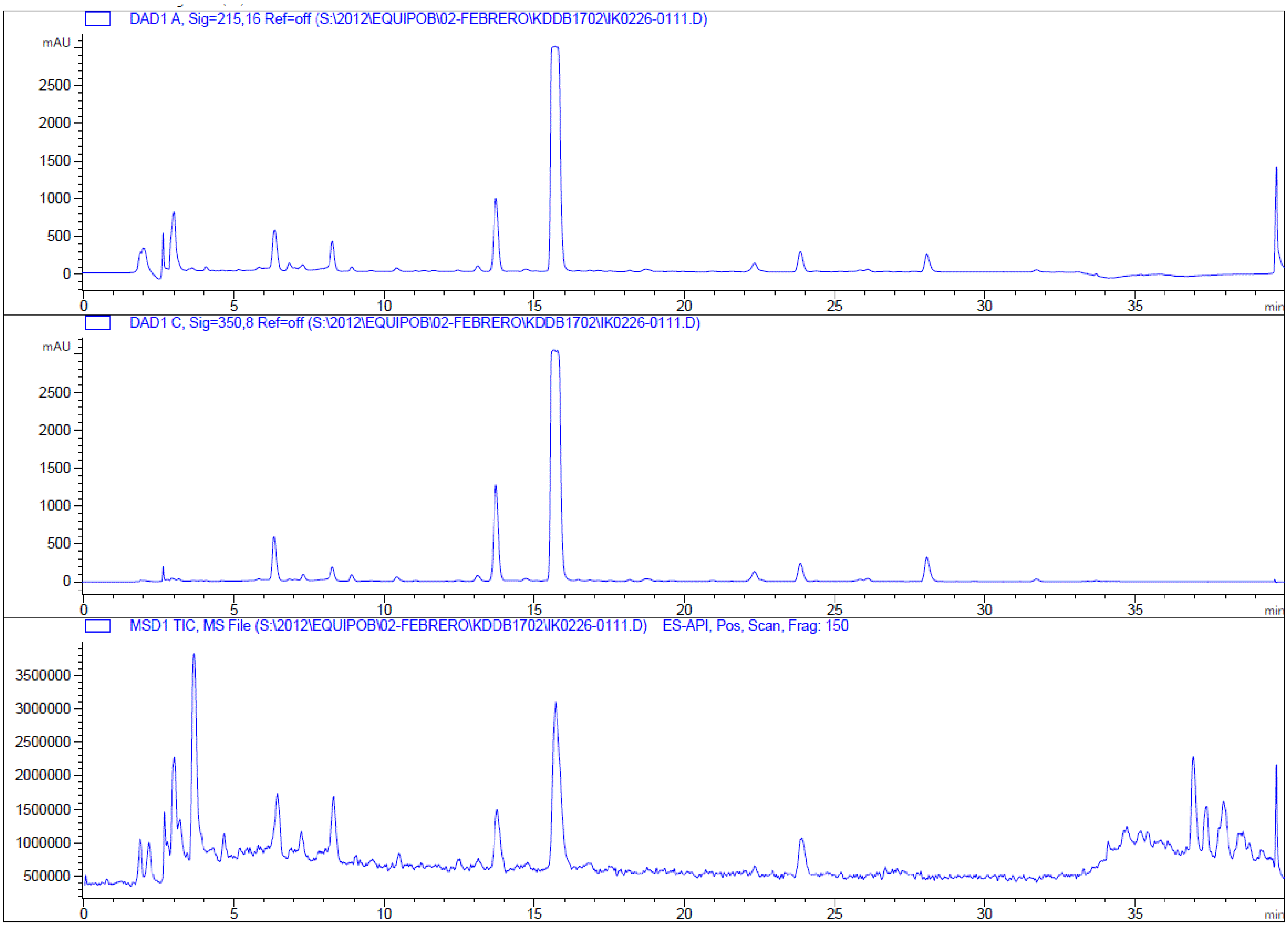

Figura S13. (+)ESIMS of hydrolysis of **1** after ozonolysis

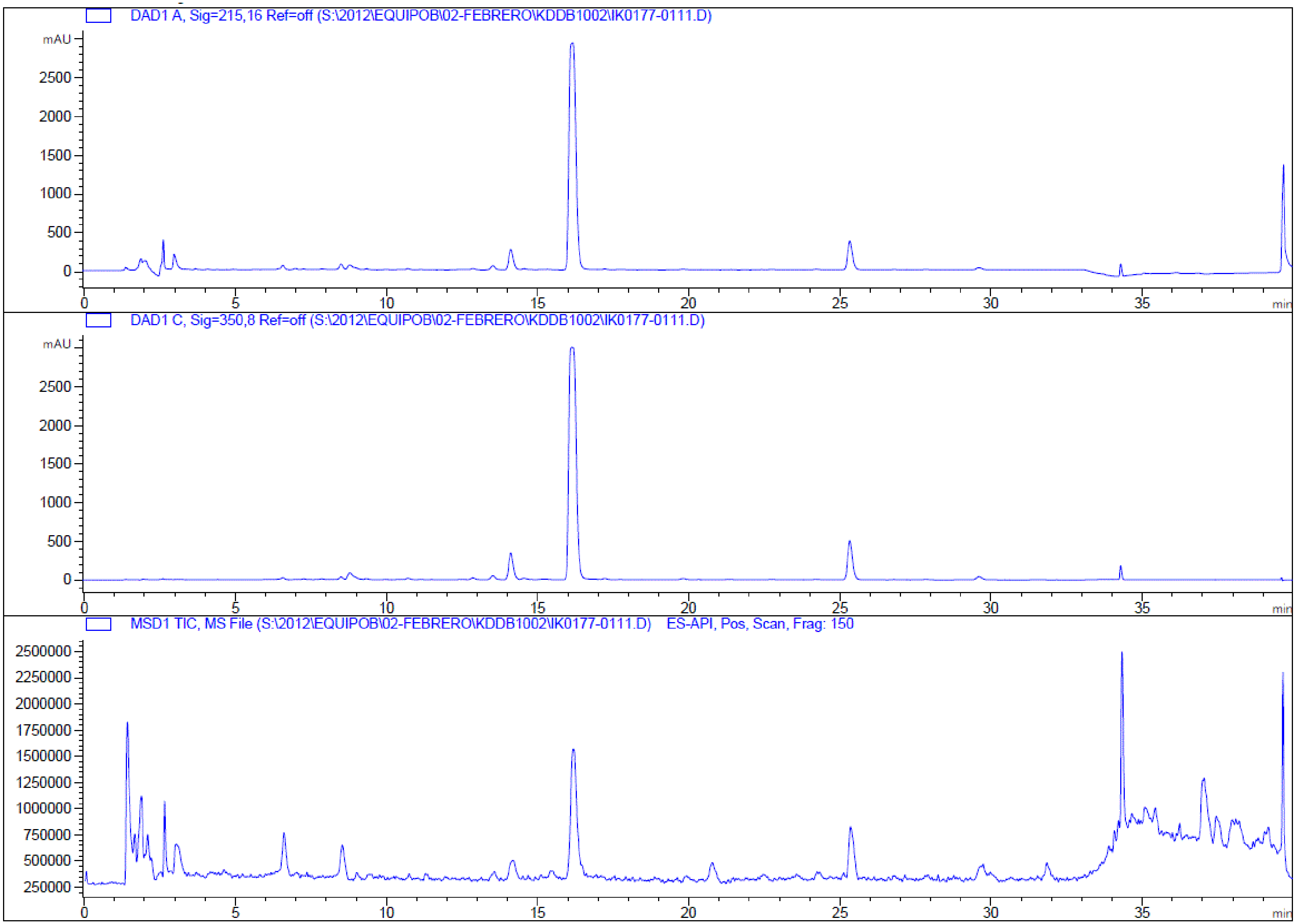

Figure S14. Extracted ion chromatogram of L-FDAA derivatives for NorVal

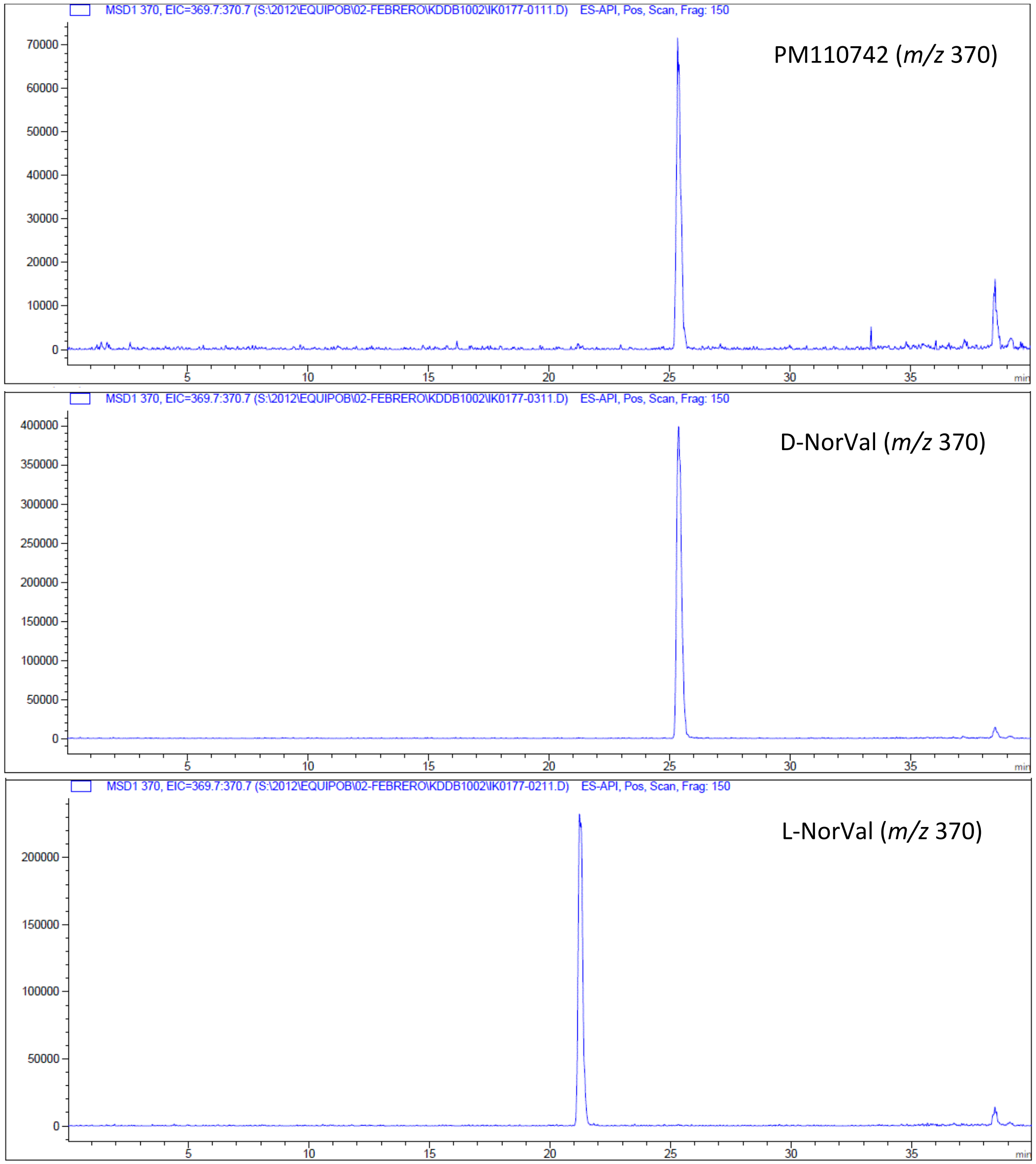

Figure S15. Extracted ion chromatogram of L-FDAA derivatives for MeCys

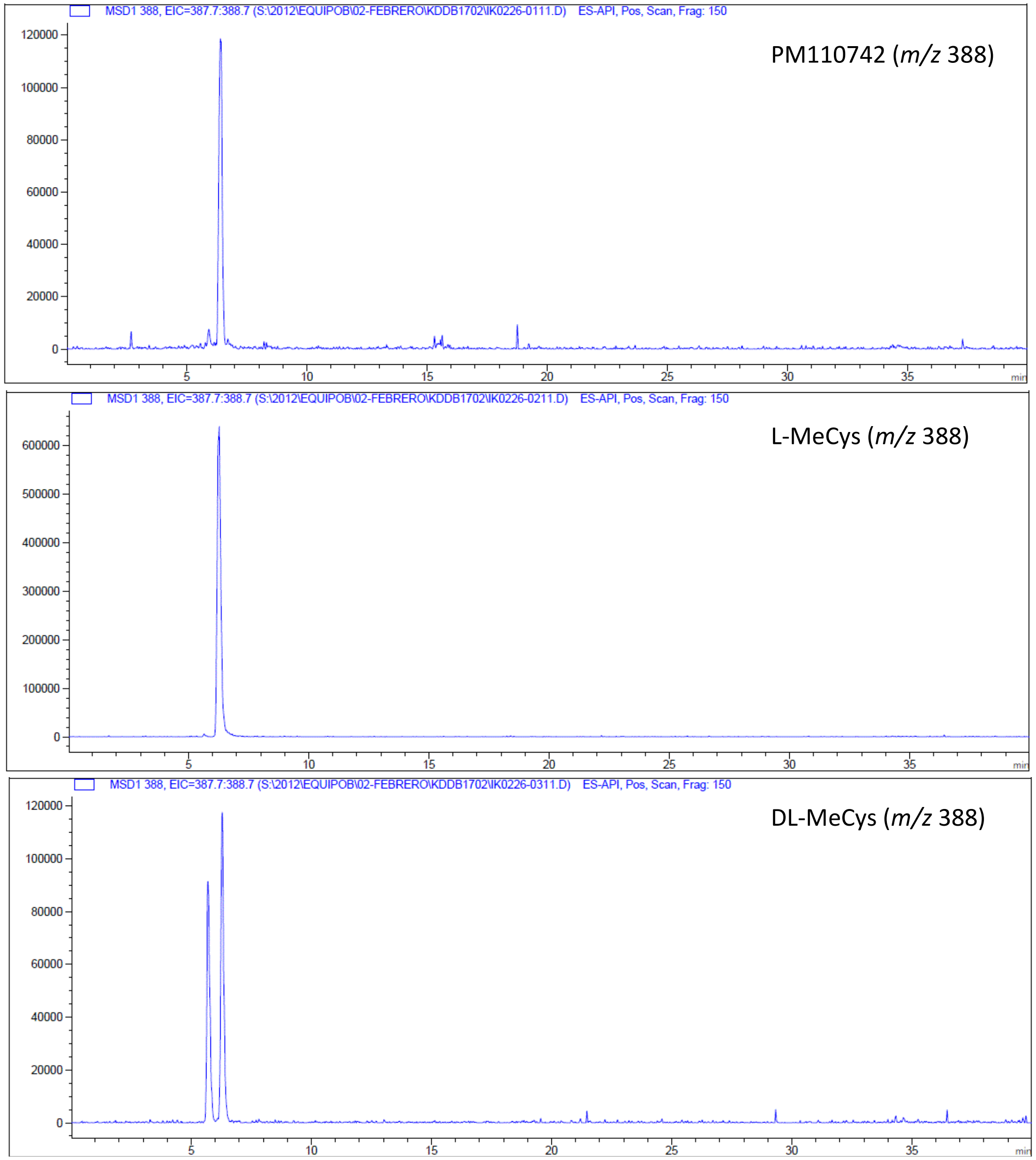

Supplement: Supplementary file 1 [file marinedrugs-22-00339-s001.zip › marinedrugs-3115033-supplementary.pdf]
